# Maternal deaths in the UK before and during the COVID‐19 pandemic

**DOI:** 10.1111/aogs.70343

**Published:** 2026-08-21

**Authors:** Allison Felker, Marian Knight

**Affiliations:** ^1^ National Perinatal Epidemiology Unit, Nuffield Department of Women's and Reproductive Health University of Oxford Oxford UK

**Keywords:** COVID‐19, direct maternal deaths, health inequalities, health services, maternal mortality, national cohort study

## Abstract

**Introduction:**

In 2020–2022, the maternal mortality rate in the United Kingdom rose to its highest level in nearly 20 years. This period coincided with the COVID‐19 pandemic, when pregnant women were at increased risk of severe infection and death. After excluding deaths directly attributed to COVID‐19, maternal mortality rates remained high, suggesting that disruptions to maternity and wider health services may have adversely affected outcomes. This study describes the patterns, causes and characteristics of maternal deaths in the UK before and during the pandemic.

**Material and Methods:**

The MBRRACE‐UK database was used to identify all maternal deaths in the UK during or up to 6 weeks after pregnancy from 2014 to 2022. The pre‐pandemic period was defined as January 2014 to February 2020 and the pandemic as March 2020 to December 2022. Data were cleaned and aggregated to permit analysis of small numbers. Maternal mortality rates and 95% confidence intervals were calculated using national maternity data as denominators. Comparisons were made between periods using chi‐square tests and mortality rate ratios (RR). Analyses were conducted in Stata 17 with statistical significance set at *p* < 0.05.

**Results:**

Maternal mortality rates were significantly higher during the pandemic compared to the pre‐pandemic period (RR 1.46, 95% CI 1.25–1.71). Rates of all direct causes of maternal deaths increased during the pandemic with statistically significant increases in deaths due to thromboembolism (RR 1.69, 95% CI 1.11–2.57) and early pregnancy‐related causes (RR 2.39, 1.02–5.61). During the pandemic, a greater proportion of women who died had mental health problems or experienced domestic abuse; a smaller proportion had pre‐existing medical problems. Persistent inequalities were observed in maternal mortality rates for women living in deprived areas or belonging to ethnic minority groups.

**Conclusions:**

These findings indicate that the effects of the COVID‐19 pandemic, including changes to service provision, were associated with increased rates of direct maternal death, underscoring the importance of maintaining equitable access to essential maternity services during future crises. The greater prevalence of mental health and social complexities amongst the women who died during the pandemic further highlights the need to improve equity in access and strengthen integrated maternity services.

## Graphical abstract

During the COVID‐19 pandemic, there was a significant increase in the rate of maternal mortality, particularly from direct causes of death. This was accompanied by a greater prevalence of mental health and social complexities amongst the women who died, underscoring the importance of maintaining equitable care access during future crises.

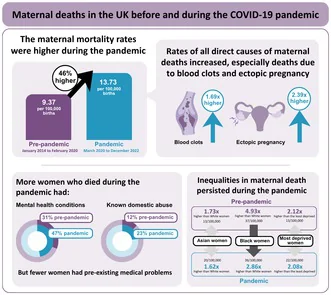

**Abbreviations**
CI: confidence interval
IMD: Index of Multiple Deprivation
MBRRACE‐UK: Mothers and Babies: Reducing Risk through Audits and Confidential Enquiries across the UK
NHS: National Health Service
RR: rate ratio

**Key message**

Maternal mortality in the UK increased significantly during the COVID‐19 pandemic, even after excluding COVID‐19 specific deaths. Increases in direct causes of death and persistent social and ethnic inequalities indicate substantial effects of service interruptions on maternal outcomes.

## 1 INTRODUCTION

Recent data show that, in the UK, the rate of maternal death during pregnancy or up to six weeks after the end of pregnancy has been steadily increasing. In 2020–2022, the maternal death rate in the UK was the highest it had been in almost 20 years.^1^

Importantly, this time period encompassed nearly 3 years following the start of the COVID‐19 pandemic. Evidence from large, multinational studies and meta‐analyses show that pregnant women are at greater risk of severe COVID‐19 infection than non‐pregnant women of reproductive age.^2^, ^3^ Pregnant women are more likely to develop severe infection, be admitted to an intensive care unit, require Extracorporeal Membrane Oxygenation (ECMO) and die from COVID‐19.^2^, ^4^ Data from UK surveillance of maternal deaths show that from January 2020 to December 2022, 38 women died from COVID‐19 infection during pregnancy or up to 6 weeks after the end of pregnancy, a rate of 1.98 per 100 000 maternities. This represents 14% of all maternal deaths from 2020 to 2022.^1^

When deaths due to COVID‐19 were excluded, UK maternal death rates from 2020 to 2022 were significantly increased from those in the three‐year period immediately preceding the COVID‐19 pandemic (2017–2019),^1^ indicating potential indirect effects of changes within maternity care and the wider health system in the UK. The National Health Service (NHS) had to make service‐wide modifications to account for staff shortages, a surge in critical care patients and reduced face‐to‐face contact. Within the UK, changes to maternity services^5^ resulted in reductions in routine antenatal appointments and fewer face‐to‐face consultations.^6^ This was also observed internationally, alongside reductions in routine antenatal screening appointments and more unscheduled attendances in emergency departments.^7^ In the UK and globally, virtual services were used widely for routine care and specialist services including for women with hypertensive disorders, diabetes, inflammatory bowel disease and for mental health care.^6^, ^7^, ^8^

Pregnant women's reported experiences of care during the pandemic reflect these changes to maternity provision with many expressing increased feelings of anxiety and confusion and a lack of autonomy.^9^, ^10^ Other women expressed concerns around privacy with virtual consultations, difficulty accessing services, inadequate service provision and a loss of trust in healthcare professionals.^10^, ^11^ In line with known inequalities in healthcare provision and maternal mortality rates,^1^ ethnic minority women's care experiences were also disproportionately impacted by the indirect changes to maternity services with many reporting incidences of bias and racism, both interpersonal and structural.^9^ These experiences suggest that the COVID‐19 pandemic, in addition to producing service interruptions, also changed the ways women engage with services and healthcare providers. However, the influence of these COVID‐19‐mandated changes to maternity services and women's health‐seeking behaviors on women's pregnancy outcomes, including maternal mortality, is not known.

The aim of this study was to describe the patterns of maternal mortality, including causes of death and the characteristics of the women who died during pregnancy and up to six weeks after the end of pregnancy in the UK before and during the COVID‐19 pandemic. As maternal mortality rates are widely considered to be a key indicator of a nation's health and the quality of the healthcare system, these data can help identify areas for potential intervention and allow lessons to be learned for maternity services during future pandemics.

## 2 MATERIAL AND METHODS

### 2.1 Data source

The Mothers and Babies: Reducing Risk through Audits and Confidential Enquiries across the UK (MBRRACE‐UK) surveillance database was used to identify all women who died in the UK during pregnancy or up to six weeks after the end of pregnancy from 2014 to 2022. Women who died before the COVID‐19 pandemic included those who died from January 2014 to February 2020, before the pandemic was declared. Women who died during the COVID‐19 pandemic included those who died from 1 March 2020 to 31 December 2022. MBRRACE‐UK is the collaboration responsible for enhanced maternal death surveillance in the UK.^12^ Maternal deaths are defined as per the World Health Organization (WHO) criteria^13^ and causes of maternal death were classified in line with International Classification of Disease Maternal Mortality (ICD‐MM) subgroups.^13^ All data held by MBRRACE‐UK are cross‐checked against national death and birth registries to ensure completeness and accuracy. After a maternal death is reported, a surveillance form is used to prospectively collect basic demographic information and clinical details about the woman, her pregnancy and her birth. MBRRACE‐UK also requests the woman's medical records that are used to supplement missing surveillance data, where available.

### 2.2 Data analyses

For the purposes of this secondary analysis, MBRRACE‐UK data were cleaned and re‐categorized into aggregate groups to permit analysis and limit potential identification of women where numbers were small. Self‐reported ethnic groups were re‐categorized into five broad categories based on the availability of denominator data from NHS Hospital Episode Statistics (HES).^14^ Socioeconomic status was determined using the woman's postcode of residence and area‐based Indices for Multiple Deprivation (IMD). A weighted ranking for Lower Layer Super Output Areas (LSOAs) was determined and women were aggregated into quintiles with IMD I being the least deprived and IMD V the most deprived areas.^15^ Pre‐existing health conditions were coded according to ICD‐10 and women were grouped into binary yes/no categories for all pre‐existing medical or mental health conditions on the basis of this coding. Recommended and minimum antenatal care was defined in accordance with guidance from the National Institute of Care Excellence (NICE).^16^

Mortality rates with 95% confidence intervals (CI) were calculated using national data on the number of maternities as the denominator. Total maternities for the periods before and during the COVID‐19 pandemic were obtained from annually reported birth data. The estimated mortality rates and 95% CI for specific ethnic and socioeconomic groups were calculated using NHS HES maternity data for England as the denominator.^14^ To adjust for temporal shifts in the maternity population over the nine‐year period, directly standardized rates for age, deprivation and ethnicity were calculated for the two time periods using the pre‐COVID‐19 period as the standard reference population. Since monthly figures on the number of maternities were not available through routine data sources, an average number of monthly maternities for 2020 was calculated using annual figures. The estimated number of maternities was doubled to calculate the number of maternities in January and February 2020 and multiplied by 10 to provide an estimate for the number of maternities between March and December 2020. These estimated figures were then added to reported annual UK maternities for 2014–2019 or 2021–22, respectively. The same process was used to calculate the denominators for different ethnic and socioeconomic groups.

The incidence of each maternal characteristic is presented as a number and percentage for each of the two groups: pre‐COVID‐19 and during the COVID‐19 pandemic. A Chi‐square test was used to compare proportions between the two groups. For mortality rates due to specific causes of death or within specific population groups (age, ethnicity, IMD quintile), the Stata command “iri” was used to calculate the mortality rate ratio and 95% CI to compare (a) different population groups and (b) women who died before and during the COVID‐19 pandemic. All data analyses were conducted using Stata 17. Statistical significance was characterized as a *p*‐value less than 0.05.

## 3 RESULTS

### 3.1 Maternal mortality rates

During the COVID‐19 pandemic, from March 2020 to December 2022, 263 women died during or within six weeks after the end of pregnancy in the UK from direct or indirect causes. Amongst 1 916 147 maternities, this represents a maternal death rate of 13.73 per 100 000 maternities (95% CI 12.12–15.49). From January 2014 to February 2020, the period preceding the COVID‐19 pandemic, 430 women died from direct or indirect causes amongst 4 587 834 maternities, a maternal mortality rate of 9.37 per 100 000 maternities (95% CI 8.51–10.30). Thus, during the COVID‐19 pandemic, there was a statistically significant increase in the overall rate of maternal death (Rate ratio (RR) 1.46, 95% CI 1.25–1.71). Statistically significant increases were observed in both the rates of maternal death due to direct causes (RR 1.54, 95% CI 1.22–1.95) and indirect causes (RR 1.40, 95% CI 1.13–1.74) (Table 1).

**TABLE 1:** Maternal mortality rates and causes of death per 100 000 maternities, during pregnancy or up to six weeks after the end of pregnancy, before and during the COVID‐19 pandemic, UK 2014–2022.

	Pre‐COVID (*n*=430^a^)			During COVID (*n*=263^b^)			Rate ratio (RR)	95% CI	*p*
	*n*	Rate per 100 000 maternities	95% CI	*n*	Rate per 100 000 maternities	95% CI			
**All direct and indirect deaths**	430	9.37	8.51–10.30	263	13.73	12.12–15.49	1.46	1.25–1.71	< 0.001
**Direct deaths**	188	4.10	3.53–4.73	121	6.31	5.24–7.55	1.54	1.22–1.95	< 0.001
Pregnancy‐related infections	24	0.52	0.34–0.78	17	0.89	0.52–1.42	1.70	0.86–3.29	0.103
Pre‐eclampsia and eclampsia	12	0.26	0.14–0.46	7	0.37	0.15–0.75	1.40	0.47–3.85	0.484
Thrombosis and thromboembolism	58	1.26	0.96–1.63	41	2.14	1.54–2.90	1.69	1.11–2.57	0.012
Amniotic fluid embolism	17	0.37	0.22–0.59	8	0.42	0.18–0.82	1.13	0.42–2.75	0.766
Early pregnancy deaths	13	0.28	0.15–0.49	13	0.68	0.36–1.16	2.39	1.02–5.61	0.030
Hemorrhage	32	0.70	0.48–0.99	15	0.78	0.44–1.29	1.12	0.56–2.13	0.702
Psychiatric causes‐Suicides	27	0.59	0.39–0.86	17	0.89	0.52–1.42	1.51	0.77–2.87	0.192
Other–direct^c^	5	0.11	0.04–0.25	3	0.16	0.03–0.46	1.44	0.22–7.38	0.619
**Indirect deaths**	242	5.27	4.63–5.98	142	7.41	6.24–8.73	1.40	1.13–1.74	0.002
Cardiac disease	91	1.98	1.60–2.44	36	1.88	1.32–2.60	0.95	0.63–1.41	0.793
Indirect infection–Influenza, pneumonia/others	18	0.39	0.23–0.62	6	0.31	0.12–0.68	0.80	0.26–2.10	0.657
COVID‐19	–	–	–	38	1.98	1.40–2.72	–	–	–
Other indirect causes	45	0.98	0.72–1.31	18	0.94	0.56–1.48	0.96	0.52–1.69	0.893
Indirect neurological conditions	56	1.22	0.92–1.59	25	1.30	0.84–1.93	1.07	0.64–1.74	0.772
Psychiatric causes–Drugs/alcohol/others	18	0.39	0.23–0.62	13	0.68	0.36–1.16	1.73	0.78–3.73	0.141
Indirect malignancies	14	0.31	0.17–0.51	6	0.31	0.12–0.68	1.03	0.32–2.84	0.934

^a^ Includes all women who died between 2014 and 2019 and 12 women who died in January 2020 or February 2020 from direct and indirect causes.

^b^ Includes only women who died between 1 March 2020 and 31 December 2022 from direct and indirect causes.

^c^ Includes women who died from complications of anesthesia, direct malignancies and other unascertained direct causes.

During the period of the pandemic, 38 women died from COVID‐19 during or within six weeks of pregnancy. When these women's deaths were excluded, the overall maternal mortality rate remained statistically significantly higher during the pandemic compared to the period before (RR 1.25, 95% CI 1.06–1.48, *p* = 0.007).

#### 3.1.1 Direct maternal deaths

Rates of all direct causes of maternal death were higher during the COVID‐19 pandemic compared to the preceding period (Table 1). The rate of death due to thrombosis and thromboembolism was statistically significantly increased during the COVID‐19 pandemic (RR 1.69, 95% CI 1.11–2.57). Neither COVID‐19 infection nor COVID‐19 vaccination were the cause of thrombosis or thromboembolism in any of the women who died. A statistically significant increase was also observed for deaths due to early pregnancy disorders, including ectopic pregnancy (RR 2.39, 95% CI 1.02–5.61) (Table 1). While not statistically significantly increased, deaths due to pregnancy‐related infections were 70% higher and deaths due to suicide were 51% higher during the COVID‐19 pandemic compared to before (Table 1).

### 3.2 Indirect maternal deaths

Most rates of indirect causes of death including cardiac disease, neurological conditions (epilepsy and stroke), medical or general surgical disorders (other indirect deaths) and hormone‐sensitive malignancies (breast, cervical and ovarian cancer) were similar in the two periods (Table 1). While not statistically significantly increased, there was an almost two‐fold increase in the rate of maternal death due to substance use during the COVID‐19 pandemic compared to the period before (RR 1.73, 95% CI 0.78–3.73, *p* = 0.141).

### 3.3 Characteristics of the women who died

The socioeconomic, medical and pregnancy‐related characteristics of the women who died during or up to 6 weeks after pregnancy in the UK before or during the COVID‐19 pandemic are shown in Table 2 and Tables S1 and S2. Of the women whose experience of domestic abuse was known, almost double the proportion of women who died during the pandemic had experienced domestic abuse (23% compared to 12% prior to the pandemic, *p* = 0.003). A greater proportion of women who died during the pandemic had social service involvement compared to women who died in the period before, but this increase was not statistically significant (26% vs. 19%, respectively, *p* = 0.059). On the basis of the information available, 7% of women who died prior to the pandemic and 9% of women who died during were considered to have multiple disadvantage (Table 2).

**TABLE 2:** Selected characteristics of the women who died during pregnancy or up to six weeks after the end of pregnancy, before and during the COVID‐19 pandemic, UK 2014–2022.

Characteristics	Pre‐COVID (*n* = 430^a^) Frequency (%)	During COVID (*n* = 263^b^) Frequency (%)	*p*
**Socioeconomic characteristics**			
Domestic abuse (prior to pregnancy/during pregnancy)			0.003
Yes	35 (12)	41 (23)	
No	254 (88)	140 (77)	
Missing	141	82	
Social service involvement			0.059
Yes	77 (19)	58 (26)	
No	320 (81)	166 (74)	
Missing	33	39	
Multiple disadvantage^c^			0.252
Yes	29 (7)	24 (9)	
No	401 (93)	239 (91)	
**Medical and health characteristics**			
Body mass index (BMI)			0.040
< 25 kg/m^2^	168 (40)	80 (32)	
≥ 25 kg/m^2^ (overweight or obese)	253 (60)	170 (68)	
Missing	9	13	
Substance use			0.520
Yes	56 (14)	36 (16)	
No	352 (86)	195 (84)	
Missing	22	32	
Mental health problems			<0.001
Yes	121 (31)	112 (47)	
No	270 (69)	125 (53)	
Missing	39	26	
Pre‐existing medical problem (excluding obesity)			0.047
Yes	284 (68)	148 (60)	
No	135 (32)	98 (40)	
Missing	11	17	
**Pregnancy‐associated characteristics**			
Parity			0.019
Nulliparous	165 (40)	81 (31)	
Multiparous	245 (60)	178 (69)	
Missing	20	4	
Received antenatal care^d^			0.003
Yes	369 (86)	202 (77)	
No	61 (14)	61 (23)	
Gestational age at booking (weeks) (amongst women who received antenatal care)			0.002
≤ 10	207 (56)	141 (70)	
11–12	71 (19)	19 (9)	
> 13	76 (21)	39 (19)	
Missing	15	3	
Received recommended antenatal care^e^ (amongst women who received antenatal care)			0.053
Yes	184 (54)	86 (46)	
No	155 (46)	103 (55)	
Missing	30	13	

^a^ Includes all women who died between 2014 and 2019 and 12 women who died in January 2020 or February 2020 from direct and indirect causes.

^b^ Includes only women who died between 1 March 2020 and 31 December 2022 from direct and indirect causes.

^c^ Multiple disadvantage is considered a score of three or more of: substance use, domestic abuse, abuse in childhood, arrival in UK in last 5 years, refugee or asylum seeker, mental health diagnosis, female genital mutilation and known learning difficulties.

^d^ Includes 11 women who died in early pregnancy (≤ 10 weeks' gestational age).

^e^ NICE recommended antenatal care: booked at 10 weeks or less and no antenatal visits missed.

During the years of the pandemic, significantly more women who died were overweight or obese though similar proportions of women were known to smoke (Table 2 and Table S2). Almost half (47%) of the women who died during the COVID‐19 pandemic had mental health problems, which is statistically significantly higher than the proportion of women who died prior to the pandemic (31%, *p* < 0.001). Significantly fewer women who died during the pandemic had pre‐existing medical problems compared to women who died before the pandemic (60% vs. 68%, *p* = 0.047) (Table 2).

Compared to women who died prior to the pandemic, significantly fewer women who died during the pandemic had received any antenatal care in their most recent pregnancy (86% vs. 77%, *p* = 0.003) (Table 2). This remained true when women who died in early pregnancy, including those who died prior to 10 weeks' gestational age or from ectopic pregnancies, were excluded from the analysis (Table S2). Amongst women who had received antenatal care, fewer women who died during the pandemic received recommended antenatal care,^16^ though this was not statistically significantly different to before the pandemic (46% vs. 54%, *p* = 0.053) (Table 2). The reduction in the number of women who received recommended antenatal care did not seem to be a consequence of later first antenatal visits, as more women had their first antenatal visit at 10 weeks' gestation or less during the pandemic (Table 2). The pregnancy outcomes for women who died before or during the COVID‐19 pandemic were largely similar (Table S2).

### 3.4 Maternal mortality in different population groups

#### 3.4.1 Age

Compared to the period before the COVID‐19 pandemic, statistically significant increases were observed in the age‐specific rates of maternal death for women aged 25–29, 30–34, 35–39 and ≥ 40 and in the overall age‐standardized rate during the pandemic (Table 3). The ratios of relative risks, comparing the relative risk of maternal death amongst different age groups before and during the pandemic, are shown in Table 4.

**TABLE 3:** Maternal mortality rates amongst different population groups before and during the COVID‐19 pandemic, 2014–2022.

	Pre‐COVID^a^				During COVID^b^				Rate ratio (RR) (comparing during the COVID‐19 pandemic with the period before)	95% CI	*p*
	Total maternities^c^	Total deaths Frequency (%)^a^	Rate per 100 000 maternities	95% CI	Total maternities^d^	Total deaths Frequency (%)^b^	Rate per 100 000 maternities	95% CI			
**Age (UK)**											
< 20	147 953	18 (4)	12.17	7.21–19.23	46 187	5 (2)	10.83	3.52–25.26	0.89	0.26–2.49	0.853
20–24	676 374	45 (10)	6.65	4.85–8.90	236 864	18 (7)	7.60	4.50–12.01	1.14	0.62–2.01	0.625
25–29	1 281 105	106 (25)	8.27	6.77–10.01	504 179	63 (24)	12.50	9.60–15.99	1.51	1.09–2.08	0.011
30–34	1 463 087	110 (26)	7.52	6.18–9.06	655 923	72 (27)	10.98	8.59–13.82	1.46	1.07–1.98	0.014
35–39	824 559	107 (25)	12.98	10.63–15.68	379 538	71 (27)	18.71	14.61–23.60	1.44	1.05–1.96	0.019
≥ 40	194 589	44 (10)	22.61	16.43–30.35	93 302	34 (13)	36.44	25.24–50.92	1.61	1.00–2.58	0.040
*Crude rate per 100 000 maternities*			9.37	8.51–10.30			13.73	12.12–15.49	1.46	1.25–1.71	<0.001
*Age‐standardized rate per 100 000 maternities*			9.37	8.51–10.30			13.36	12.33–14.46	1.43	1.26–1.62	<0.001
**IMD Quintiles (England only)** ^e^											
I (Least deprived/highest 20%)	548 426	33 (10)	6.02	4.14–8.45	232 197	24 (11)	10.34	6.62–15.38	1.72	0.97–3.00	0.048
II	618 313	35 (11)	5.66	3.94–7.87	263 293	25 (11)	9.50	6.14–14.02	1.68	0.96–2.88	0.053
III	697 009	56 (17)	8.03	6.07–10.43	290 497	49 (22)	16.87	12.48–22.30	2.05	1.36–3.06	<0.001
IV	835 569	81 (24)	9.69	7.70–12.05	335 163	42 (19)	12.53	9.03–16.94	1.29	0.87–1.90	0.181
V (Most deprived/lowest 20%)	1 001 278	128 (38)	12.78	10.67–15.20	390 240	84 (38)	21.53	17.17–26.65	1.68	1.26–2.23	<0.001
*Crude rate per 100 000 maternities*			9.00	8.06–10.02			14.82	12.94–16.89	1.65	1.38–1.96	<0.001
*Deprivation‐standardized rate per 100 000 maternities*			9.00	8.06–10.02			14.95	13.72–16.24	1.66	1.45–1.91	<0.001
**Ethnic group (England only)**											
White (inc. not known)	3 050 961	231 (63)	7.57	6.63–8.61	1 218 721	152 (66)	12.47	10.57–14.62	1.65	1.33–2.03	<0.001
Asian	397 457	52 (14)	13.08	9.77–17.15	192 981	39 (17)	20.21	14.37–27.63	1.55	0.99–2.39	0.043
Black	168 944	63 (17)	37.29	28.66–47.71	78 510	28 (12)	35.66	23.70–51.54	0.96	0.59–1.52	0.856
Chinese/others	153 917	11 (3)	7.15	3.57–12.79	67 135	6 (3)	8.94	3.28–19.45	1.25	0.38–3.69	0.652
Mixed	64 888	10 (3)	15.41	7.39–28.34d	34 899	6 (3)	17.19	6.31–37.42	1.12	0.33–3.39	0.820
*Crude rate per 100 000 maternities*			9.57	8.61–10.60			14.51	12.70–16.50	1.52	1.28–1.79	<0.001
*Ethnicity‐standardized rate per 100 000 maternities*			9.57	8.61–10.60			14.23	13.06–15.48	1.49	1.30–1.70	<0.001

^a^ Includes all women who died between 2014 and 2019 and 12 women who died in January 2020 or February 2020 from direct and indirect causes in the UK (age group) or England (IMD quintile and ethnic group).

^b^ Includes only women who died between 1 March 2020 and 31 December 2022 from direct and indirect causes in the UK (age group) or England (IMD quintile and ethnic group).

^c^ Includes all maternities in the UK (age group) or England (IMD quintile and ethnic group) from 1 January 2014–29 February 2020.

^d^ Includes all maternities in the UK (age group) or England (IMD quintile and ethnic group) from 1 March 2020–31 December 2022.

^e^ IMD Quintile is not known for 41 women.

**TABLE 4:** Comparing the rate ratio of maternal death amongst different population groups before and during the COVID‐19 pandemic, 2014–2022.

	Pre‐COVID^a^		During COVID^b^		Ratio of the rate ratios (RRR) (comparing rate ratio during the COVID‐19 pandemic with the period before)	95% CI	*p*
	Rate ratio (RR)	95% CI	Rate ratio (RR)	95% CI			
**Age (UK)**							
< 20	1.47	0.84–2.44	0.87	0.27–2.13	0.59	0.19–1.89	0.377
20–24	0.80	0.55–1.15	0.61	0.34–1.04	0.76	0.39–1.49	0.428
25–29	1 (ref)	–	1 (ref)	–	–	–	–
30–34	0.91	0.69–1.20	0.88	0.62–1.25	0.97	0.62–1.51	0.883
35–39	1.57	1.19–2.07	1.50	1.05–2.14	0.96	0.61–1.50	0.843
≥ 40	2.73	1.88–3.92	2.92	1.86–4.49	1.07	0.60–1.90	0.818
**IMD Quintiles (England only)** ^c^							
I (Least deprived/highest 20%)	1 (ref)	–	–	1 (ref)	–	–	–
II	0.94	0.57–1.56	0.92	0.50–1.68	0.98	0.45–2.15	0.958
III	1.33	0.85–2.12	1.63	0.98–2.78	1.23	0.61–2.45	0.565
IV	1.61	1.06–2.49	1.21	0.72–2.09	0.75	0.38–1.49	0.413
V (Most deprived/lowest 20%)	2.12	1.44–3.22	2.08	1.31–3.43	0.98	0.52–1.84	0.953
**Ethnic group (England only)**							
White (inc. not known)	1 (ref)	–	1 (ref)	–	–	–	–
Asian	1.73	1.25–2.34	1.62	1.11–2.32	0.94	0.58–1.52	0.790
Black	4.93	3.67–6.53	2.86	1.84–4.30	0.58	0.35–0.97	0.038
Chinese/others	0.94	0.46–1.72	0.72	0.26–1.60	0.77	0.25–2.35	0.642
Mixed	2.04	0.96–3.81	1.38	0.50–3.07	0.68	0.22–2.11	0.502

^a^ Includes all women who died between 2014 and 2019 and 12 women who died in January 2020 or February 2020 from direct and indirect causes in the UK (age group) or England (IMD quintile and ethnic group).

^b^ Includes only women who died between 1 March 2020 and 31 December 2022 from direct and indirect causes in the UK (age group) or England (IMD quintile and ethnic group).

^c^ IMD Quintile is not known for 41 women.

#### 3.4.2 Socioeconomic status

In both periods, 38% of the women who died were living in the most deprived areas (Table 3) and had a two‐fold increased risk of death compared to women living in the least deprived areas (Table 4). This disparity in the risk of death remained unchanged by the COVID‐19 pandemic (RR 0.98, 95% CI 0.52–1.84, *p* = 0.953) but the rate of maternal death in all but one IMD quintile was significantly higher during the pandemic compared to the period before. This includes a significant increase in mortality in the group used as the baseline (IMD I) (Table 3). After adjusting for deprivation in the underlying population, the overall mortality rate during the COVID‐19 pandemic remained statistically significantly increased from the period before (Table 3).

#### 3.4.3 Ethnicity

During COVID‐19, the maternal mortality rates for women from most ethnic backgrounds were increased from pre‐pandemic rates. This was also true of the overall ethnicity‐standardized maternal mortality rate (Table 3). This increase in the rate of maternal death did not, however, correspond to a similar increase in the relative risk of mortality for women from specific ethnic groups (Table 4). Compared to White women, Asian women were at a 1.7 times higher risk of death before the pandemic (RR 1.73, 95% CI 1.25–2.34) and 1.6 times higher risk of death during the pandemic (RR 1.62, 95% CI 1.11–2.32). Notably, while Black women continued to experience substantial inequalities in maternal mortality, the magnitude of this inequality statistically significantly decreased during the pandemic compared to the period before (Table 4).

## 4 DISCUSSION

This study found statistically significantly higher rates of maternal death during the pandemic than in the period prior to the pandemic. This remained true when deaths directly attributed to COVID‐19 were excluded and when rates were independently standardized for age, deprivation and ethnicity. Increases were most notable for direct causes of maternal death; all cause‐specific rates of direct maternal death were higher during the pandemic than in the period before. A higher proportion of women who died during the pandemic had elements of disadvantage including mental health diagnoses and experiences of domestic abuse, and inequalities in maternal death relating to age, level of deprivation and ethnicity also persisted during the pandemic. These findings highlight the need for future pandemic planning within maternity services to prioritize equitable access to care with targeted support for vulnerable women to mitigate indirect impacts of changes to health services.

The rates of all direct causes of maternal death, which are largely thought to be preventable, were increased during the COVID‐19 pandemic compared to the period before. For maternal deaths due to thrombosis and thromboembolism and early pregnancy‐related causes, these increases were statistically significant. While changes in the underlying maternity population, including a higher prevalence of obesity, may have contributed to an increase in these maternal deaths, findings from a recent MBRRACE‐UK confidential enquiry showed evidence of pandemic‐related service interruptions that may also have contributed.^1^ This was primarily seen in pre‐hospital care, where delayed ambulance response times and transfer to hospital, impacted care during the pandemic. In some instances, this lead to delayed or missed management of acute conditions such as ruptured ectopic pregnancies.^1^ This finding is supported by a recent global meta‐analysis, which demonstrated six‐fold higher odds of surgical treatment for ectopic pregnancies during the pandemic, likely reflecting increased rates of rupture due to delayed presentation, diagnosis and intervention.^3^ Service interruptions were also reflected in changes to routine antenatal care amongst the women who died. Results from this study show that significantly fewer women who died during COVID‐19 received antenatal care, a finding that remained true when women who died in early pregnancy were excluded from analysis. Widespread changes to antenatal care appointments were reported across the UK during the pandemic, including reductions in the number of appointments and the mode, with many appointments moving remotely.^6^ While it is possible that the level of disruption caused by changes to service provision varied depending on the phase of the pandemic, and thus may have impacted temporal changes in mortality within the pandemic years (2020–2022), most interruptions persisted across all phases of the pandemic and are still impacting care today. For instance, many antenatal appointments are still conducted virtually^17^ despite studies showing that reduced face‐to‐face clinical assessment can result in missed recognition or underappreciation of concerning symptoms.^1^, ^18^, ^19^ During COVID‐19, women also reported long intervals between antenatal appointments and high levels of cancellations,^20^ which is reflective of broader pandemic‐era issues around workface shortages and staff reallocation from maternity services to other clinical areas.^1^, ^18^, ^19^ These capacity restraints meant that many women did not receive appropriate multidisciplinary care, senior oversight or specialist care during the pandemic,^1^, ^12^, ^18^, ^19^ which may have impacted outcomes. These shortages and capacity issues were reported across the years of the pandemic (2020–2022) and are still impacting the NHS to date.

In contrast to the increase in direct maternal deaths, this study found no significant difference in the rate of maternal death from any indirect condition during the pandemic, and far fewer women who died during the pandemic had pre‐existing medical conditions (excluding obesity) compared with women who died prior to the pandemic. However, it is unclear if these findings are indicative of changes in the characteristics and risks of the maternity population, or a consequence of how maternity services were modified and managed immediately preceding and during the pandemic. Initiatives put forward in the NHS's Long Term Plan, published in 2019, aimed for improved provision of networked care for pregnant women with medical problems.^21^ This also included an increased focus on obstetric medicine through training of more obstetric physicians and the development of maternal medicine networks. Evidence from the UK and larger, international meta‐analyses also suggests that most COVID‐era modifications to maternity care did not impact specialist services to the same degree as routine, low‐risk appointments. Rather, high‐risk women with medical comorbidities typically continued with usual appointment schedules.^6^ This sustainment in care for high‐risk women, including those with pre‐existing medical conditions, may help explain why the rate of indirect deaths was unchanged during the pandemic. However, these findings highlight a potential inequity in access to antenatal services during the pandemic. While the sustained service provision for high‐risk women is positive, future pandemic planning must safeguard all aspects of maternity care, including routine antenatal care pathways. It is imperative that planning for future emergencies considers potential workforce shortages, especially in pre‐hospital and emergency obstetric settings to manage acute, potentially preventable conditions such as thrombosis and ectopic pregnancy.

This study found that the prevalence of mental health problems was higher amongst women who died during the pandemic compared to the period before. The rates of death due to psychiatric causes, including suicide and substance use, were also 50% and 73% higher, respectively, during the COVID‐19 pandemic; however, these figures are an underestimation of the true number of women who died from mental health‐related conditions during the pandemic, as the vast majority of maternal deaths due to psychiatric causes occur in the late perinatal period after women have transitioned out of maternity care.^22^ The methodological approaches used in this study cannot determine whether the increases in the prevalence of mental health conditions or rates of death from psychiatric causes are reflective of actual risk or in changes to the underlying maternity population during the pandemic. Other studies showed an increased prevalence of perinatal mental health disorders during the COVID‐19 pandemic,^17^, ^23^ which may be reflective of the women who died. However, despite this increased prevalence and higher demand for perinatal mental health services during the pandemic, evidence suggests that referral rates, including for inpatient services, declined^8^ and admissions to mother and baby units were shorter on average.^24^ Challenges were also noted in the capacity of specialist perinatal mental health services to accept referrals^22^ and staff reported concerns about their ability to detect signs of mental illness and properly risk assess women during remote consultation.^8^ This suggests that changes to these services, as well as an increase in the prevalence of perinatal mental health disorders in the overall maternity population, may have contributed to the observed increase in the rates of maternal deaths due to psychiatric causes during the pandemic. For women who are engaged with mental health services before pregnancy, it is essential that care continues throughout the perinatal period. Women should be routinely monitored for any new or worsening symptoms and escalation plans should be in place for any potential stressors or destabilizing circumstances, such as those introduced by pandemics or public health emergencies.

Similar to the increase in mental health conditions, there was a significantly higher proportion of women who died during the pandemic who had known domestic abuse compared to the period before. Non‐significant increases were also observed in the proportion of women with social service involvement. Women experiencing multiple disadvantages, including mental health issues, substance use, domestic abuse and social service involvement, are consistently overrepresented in maternal mortality figures^22^ and often experience barriers accessing care as they are required to engage with many different supports and services throughout their pregnancy and postnatal period.^22^, ^25^ Reports have highlighted the need to integrate maternity care, primary care, and support services to improve information sharing and deliver holistic, personalized care.^22^, ^25^ Efforts to deliver such coordinated care were impacted by the COVID‐19 pandemic as repeated reconfiguration of maternity services affected continuity of carer, patient‐clinician relationships and trust, which are essential for engagement and disclosure of sensitive information, such as experiences of domestic abuse.^22^, ^25^, ^26^ However, similar to the findings on mental health conditions, the methodology used in this study cannot accurately determine whether the increased prevalence of domestic abuse, and other social complexities, amongst the women who died during the pandemic is a result of changes to care pathways or a consequence of changes in overall prevalence. Findings on the number of reported domestic abuse incidences during the pandemic are mixed with some studies showing increased incidences,^27^ as is reflected in this study, and others showing no change or a reduction.^28^, ^29^ In other studies, decreased rates of disclosure and referrals for support services within primary care services were contrasted with higher utilization of domestic violence hotlines, indicating a disconnect between the need for, and access to, support services.^29^ As pregnancy and the postnatal period are well‐recognized periods of vulnerability with regards to domestic abuse,^30^ the provision of support for women experiencing domestic abuse must remain a priority. This includes a need to consider the exacerbation of harm through lockdown measures that impact women's ability to leave abusive environments.^29^, ^31^

Another element of potential vulnerability that must be considered in the context of this study and future pandemic planning is the persistent inequalities in maternal mortality for women living in the most deprived areas or belonging to Black or Asian ethnic groups. In line with previous MBRRACE‐UK reports, this study showed that women living in the most deprived areas had a two‐fold greater risk of maternal death both before and during the COVID‐19 pandemic compared to women living in the least deprived areas. This was also the case for women from Asian ethnic backgrounds when compared to White women. Interestingly, the rate ratio of maternal death for Black women compared to White women was statistically significantly lower during the COVID‐19 pandemic compared to the period before; however, this decrease should not be misinterpreted as progress as the rate of maternal mortality for Black women during the pandemic was not statistically significant lower than the rate prior to the pandemic. Rather, a corresponding increase in the rate of maternal death for White women, used as the baseline group to calculate rate ratios, may have concealed persistent inequalities amongst Black women. Indeed, surveys investigating Black and other ethnic minority women's experiences of maternity care in the UK during the pandemic highlighted ongoing instances of racism, discrimination and cultural insensitivity.^9^, ^32^ Evidence also showed that Black and Asian women faced inequalities in the recognition and treatment of complex conditions,^33^ were less likely to be asked about their emotional wellbeing or mental health^34^ and were more likely to have fewer antenatal appointments compared to White women.^35^ These differences in how women from ethnic minority backgrounds engage with and access care, and how they are treated within maternity services, all impact maternal outcomes. It is important to consider these experiences, as well as their intersectionality with physical and social determinants of health in any future pandemic planning. There are many new initiatives in the UK aimed at reducing inequalities in maternal health outcomes; however, specific approaches that consider the unique needs of ethnic minority groups are needed.

While other studies have investigated the rates of maternal deaths specifically due to COVID‐19 infection, this study is the first study to investigate the impact of the COVID‐19 pandemic on all maternal deaths in the UK and explore changes in the characteristics of the women who died during this period. A major strength of this study is the use of national surveillance data, which allowed for inclusion of all women who died during or up to six weeks after pregnancy from 2014 to 2022. The completeness of this sample and the breadth of the data collected for each woman allowed for extensive evaluation that would not be possible using routine data sources.

Another strength of this study is the presentation of maternal mortality rates for IMD quintiles, age groups and ethnicities based on specific denominator populations. This allowed for investigation of the risk of maternal death in relation to the underlying maternity population and accounted for any changes in the underlying population that may have occurred during the COVID‐19 pandemic. It also permitted investigation of any changes in maternal mortality inequalities by comparing the specific risk of maternal death between the two time periods. However, we note that the level of deprivation used in this study represents a population‐based measure based on the woman's area of residence and is not based on individual‐level measures, which are not available in routine data. Thus, the risk of maternal death related to IMD quintile may not be representative of the actual level of risk for each woman included in the group. We also note that the denominator figures for 2020 are based on estimated maternity figures as monthly numbers of maternities (including both live births and stillbirths) were not available from routine sources of data. However, we do not believe that these numbers are affected by seasonality as monthly data on live births, which are available, show a similar number of average births in January and February 2020 as for the remainder of the year.^36^ As the denominator is based on finalized annual figures, pandemic‐era delays in birth registration would also not affect these numbers.

Our study also had several limitations relating to the limitations of small numbers. As maternal mortality is a rare event in the UK, the numbers are small and, thus, the power of statistical analyses is low. This is especially true for subgroup analysis, which sometimes involved very small numbers and may require cautious interpretation of any observed differences. It was also not possible to perform adjusted analyses for potential confounding factors such as age, ethnicity, socioeconomic deprivation, or BMI due to small numbers and limited data available on such characteristics in denominator maternity populations. Small numbers also prevented a more detailed investigation into the different phases of the COVID‐19 pandemic and the potential impact of changing restrictions and lockdown measures on access to healthcare and maternal mortality rates.

Other limitations relate to the completeness of data within the maternal mortality surveillance dataset. These data are collected from the woman's maternity records and clinical notes and are dependent on the information recorded within these records. Missing data was particularly apparent for elements relating to social risk such as domestic abuse and social service involvement. Current UK guidelines are inconclusive about what social risk factors women should be asked about during pregnancy and how often these discussions should occur. As such, these variables are often amongst the most poorly recorded in maternity records.

## 5 CONCLUSION

The findings of this study show the impact of pandemic era changes to maternity services and widespread health system disruption on maternal mortality in the UK and provide valuable lessons for future emergency preparedness. Significant increases in maternal deaths were observed during the years of the COVID‐19 pandemic in the UK, particularly from direct causes of death requiring early recognition of symptoms and acute intervention. Cause‐specific rates of indirect deaths were not increased during the pandemic suggesting that access to specialist care may not have been impacted to the same extent as routine or emergency services. Efforts for future pandemic planning must ensure that all care pathways are equally resilient. The greater proportion of women with known mental health conditions or social risk factors, such as domestic abuse, amongst women who died during the pandemic emphasizes the need for integrated, cross‐sector approaches to care that sustain provision of perinatal mental health, safeguarding services and community supports in times when service reconfiguration and social distancing measures are imposed. Persistent inequalities in maternal mortality highlight the need for continued, targeted efforts to improve access to care for ethnic minority women and women living in the most deprived areas. As the healthcare system continues to recover from the effects of the pandemic, it is important to recognize the variability in service provision for maternity care and ensure that future pandemic planning embeds adaptable, equitable and coordinated approaches so that women do not fall through the gaps, especially if they are amongst the most vulnerable.

## AUTHOR CONTRIBUTIONS

AF and MK conceptualized and designed the study and had access to and verified the data reported in the manuscript. AF undertook the data analysis and generated the figures and tables. AF wrote the original draft and MK contributed to review and editing. The corresponding author attests that all listed authors meet authorship criteria and that no others meeting the criteria have been omitted.

## FUNDING INFORMATION

Healthcare Quality Improvement Partnership (HQIP) (HQIP NCA 2141). NIHR Senior Investigator Award (NIHR201333).

## CONFLICT OF INTEREST STATEMENT

AF and MK are part of the Maternal, Newborn and Infant Clinical outcome Review Programme (MNI‐CORP), delivered by MBRRACE‐UK and commissioned by the Healthcare Quality Improvement Partnership (HQIP) on behalf of NHS England and the governments of Wales, Northern Ireland, Jersey, Guernsey, Scotland and the Isle of Man as part of the National Clinical Audit and Patient Outcomes Programme. MK is also funded through an NIHR Senior Investigator Award. No authors have other relationships or activities that could appear to have influenced the submitted work. The views expressed are those of the authors and not necessarily those of the NIHR, the Department of Health and Social Care or HQIP. The study funders (NIHR and HQIP) had no role in study design, data collection, data analysis, data interpretation, or writing of the report, or in the decision to submit the report for publication.

## ETHICS STATEMENT

Identifiable information about maternal deaths occurring in England and Wales was collected without consent with approval of the Secretary of State for Health and Social Care under Section 251 of the NHS Act 2006 (15/CAG/0119). Data from Scotland were collected by MBRRACE‐UK without consent with approval from the Public Benefit and Privacy Panel for Health and Social Care (PBPP 2223–0101). Identifiable information was not collected from Northern Ireland.

## Supporting information


**Table S1:** Detailed socioeconomic characteristics of the women who died during pregnancy or up to six weeks after pregnancy, before and during the COVID‐19 pandemic, UK 2014–2022.
**Table S2:** Detailed medical and pregnancy‐related characteristics of the women who died during pregnancy or up to six weeks after pregnancy, before and during the COVID‐19 pandemic, UK 2014‐2022.

## Data Availability

Data may be requested through the Healthcare Quality Improvement Partnership (HQIP): https://www.hqip.org.uk/national‐programmes/accessing‐ncapop‐data/. Permission to use the MBRRACE‐UK dataset for this analysis was provided by HQIP following the approval of an extended analysis and output request.
