# Supplementary material for: Maternal deaths in the UK before and during the COVID‐19 pandemic
Source: Acta Obstet Gynecol Scand. 2026 Aug 21:10.1111/aogs.70343. Online ahead of print. doi: 10.1111/aogs.70343 (PMC13494486; doi:10.1111/aogs.70343)
Supplement: Supplementary file 1 [file AOGS-9999-0-s001.docx]

**Table S1: Detailed socioeconomic characteristics of the women who died during pregnancy or up to six weeks after pregnancy, before and during the COVID-19 pandemic, UK 2014-2022**

| Characteristics | Pre-COVID  (n=430^a^)  Frequency (%) | During COVID  (n=263^b^)  Frequency (%) | P-value |
| --- | --- | --- | --- |
| Partner |  |  | <0.001 |
| Yes | 358 (84) | 240 (95) |  |
| No | 66 (16) | 13 (5) |  |
| Missing | 6 | 10 |  |
| Living arrangements |  |  | 0.016 |
| With partner/family | 345 (86) | 213 (93) |  |
| Living alone | 55 (14) | 17 (7) |  |
| Missing | 30 | 33 |  |
| Socioeconomic status (Occupational classification) |  |  | 0.019 |
| Employed (Either woman or partner) | 259 (76) | 184 (84) |  |
| Unemployed (Both) | 83 (24) | 35 (16) |  |
| Missing | 88 | 44 |  |
| Born in the UK |  |  | 0.230 |
| Yes | 285 (73) | 185 (78) |  |
| No | 103 (27) | 53 (22) |  |
| Missing | 42 | 25 |  |
| Region of birth |  |  | 0.322 |
| United Kingdom | 285 (66) | 185 (70) |  |
| Eastern Europe | 15 (3) | 9 (3) |  |
| Asia | 35 (8) | 27 (10) |  |
| Africa | 37 (9) | 14 (5) |  |
| Other (Western Europe, Australia, North/South/Central America) | 16 (4) | 3 (1) |  |
| Missing | 42 | 25 |  |
| Able to speak/understand English |  |  | 0.640 |
| Yes | 410 (96) | 249 (97) |  |
| No | 18 (4) | 9 (3) |  |
| Missing | 2 | 5 |  |
| Ethnicity |  |  | 0.685 |
| White European | 280 (65) | 175 (67) |  |
| Indian | 19 (4) | 13 (5) |  |
| Pakistani | 18 (4) | 18 (7) |  |
| Bangladeshi | 9 (2) | 3 (1) |  |
| Other Asian | 13 (3) | 7 (3) |  |
| Black Caribbean | 18 (4) | 8 (3) |  |
| Black African | 39 (9) | 17 (6) |  |
| Others/ Mixed | 25 (6) | 17 (6) |  |
| Missing | 9 | 4 |  |
| History of abuse as a child |  |  | 0.745 |
| Yes | 14 (9) | 14 (10) |  |
| No | 140 (91) | 123 (90) |  |
| Missing | 276 | 126 |  |

^a^ Includes all women who died between 2014-2019 and 12 women who died in January 2020 or February 2020 from direct and indirect causes

^b^ Includes only women who died between 1 March 2020 – 31 December 2022 from direct and indirect causes

**Table S2: Detailed medical and pregnancy-related characteristics of the women who died during pregnancy or up to six weeks after pregnancy, before and during the COVID-19 pandemic, UK 2014-2022**

| Characteristics | Pre-COVID  (n=430^a^)  Frequency (%) | During COVID  (n=263^b^)  Frequency (%) | P-value |
| --- | --- | --- | --- |
| Smoking during pregnancy |  |  | 0.179 |
| Yes | 114 (30) | 58 (25) |  |
| No | 264 (70) | 173 (75) |  |
| Missing | 52 | 32 |  |
| Previous caesarean section (amongst parous women) |  |  | 0.375 |
| Yes | 85 (35) | 67 (39) |  |
| No | 157 (65) | 103 (61) |  |
| Missing | 3 | 8 |  |
| Number of previous caesarean section (amongst women who had a previous caesarean section) |  |  | 0.922 |
| 1 | 59 (69) | 47 (70) |  |
| ≥2 | 26 (31) | 20 (30) |  |
| Received antenatal care (excluding deaths in early pregnancy)^c^ |  |  | 0.004 |
| Yes | 365 (89) | 197 (80) |  |
| No | 47 (11) | 48 (20) |  |
| Received minimum level of antenatal care^d^ (among women who received antenatal care) |  |  | 0.877 |
| Yes | 248 (74) | 140 (75) |  |
| No | 86 (26) | 47 (25) |  |
| Missing | 35 | 15 |  |
| Any missed antenatal visits (amongst women who booked antenatal care at 10 weeks’ gestation or less) |  |  | 0.778 |
| Yes | 34 (16) | 27 (19) |  |
| No | 155 (75) | 101 (72) |  |
| Missing | 18 | 13 |  |
| Was woman undelivered at death |  |  | 0.767 |
| Yes | 123 (29) | 78 (30) |  |
| No | 307 (71) | 185 (70) |  |
| Place of delivery |  |  | 0.774 |
| Hospital | 244 (81) | 145 (82) |  |
| Outside of hospital (home, ambulance or emergency department) | 56 (19) | 31 (18) |  |
| Missing | 130 | 87 |  |
| Was delivery by caesarean section (amongst women who delivered >20 weeks’ gestation) |  |  | 0.119 |
| Yes | 183 (67) | 114 (74) |  |
| No | 91 (33) | 40 (26) |  |
| Other maternal complication |  |  | <0.001 |
| Yes | 102 (24) | 15 (6) |  |
| No | 323 (75) | 244 (93) |  |
| Missing | 5 | 4 |  |
| Critical care admission |  |  | 0.085 |
| Yes | 166 (39) | 119 (45) |  |
| No | 264 (61) | 144 (55) |  |

^a^ Includes all women who died between 2014-2019 and 12 women who died in January 2020 or February 2020 from direct and indirect causes

^b^ Includes only women who died between 1 March 2020 – 31 December 2022 from direct and indirect causes

^c^ Does not include 36 women who died at 10 weeks’ gestational age or less or who had ectopic pregnancies

^d^ NICE recommended antenatal care: booked at 10 weeks or less and no antenatal visits missed. Minimum level of care: booked at less than 13 weeks and 3 or fewer antenatal visits missed.
